# Supplementary material for: Neurophysiological evidence of sensory prediction errors driving speech sensorimotor adaptation
Source: bioRxiv. 2023 Nov 6:2023.10.22.563504. Preprint. [Version 2] doi: 10.1101/2023.10.22.563504 (PMC10634734; doi:10.1101/2023.10.22.563504)
Supplement: Supplementary file 1 [file NIHPP2023.10.22.563504V2-supplement-1.pdf]

bioRxiv preprint doi: https://doi.org/10.1101/2023.10.22.563504; this version posted November 6, 2023. The copyright holder for this preprint (which was not certified by peer review) is the author/funder, who has granted bioRxiv a license to display the preprint in perpetuity. It is made available under aCC-BY 4.0 International license.

Supplemental Information 1: Repeated adaptation session

Unlike arm reaching adaptation studies showing that re-experiencing adaptation results in savings (e.g., Huang et al., 2011) or attenuation in case of implicit adaptation (Avraham et al., 2021), we did not find any significant changes on the repeated adaptation session (2nd session, see Fig. S1A). The un-normalized baseline phase (before normalizing the baseline phase to 0 Hz) showed no significant difference across the two sessions, $t(13) = -1.985$, $p = 0.069$ (Fig. S1B, left), though the p-value was close to 0.05 due to two individuals whose baseline was much lower in the 2nd session than the 1st session. Nonetheless, there was no clear trend on whether these individuals learned more or less in the 2nd session. Indeed, the normalized adaptation data clearly shows that both the early and late phase data in the repeated adaptation session was not different from the initial adaptation (Fig. S1B, right).

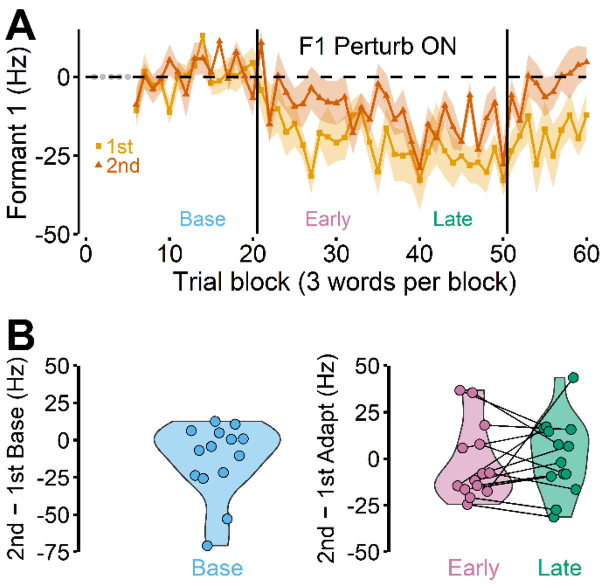

Fig. S1. A: Adaptation did not differ between the first and second sessions. B: The overall baseline also did not change in most participants (left). There were two participants whose baseline in the second session was reduced by more than 50 Hz, but as a group the baseline did not differ between the two sessions. Overall, participants as a group, there was no sign of savings or attenuation in the second session (right).

bioRxiv preprint doi: https://doi.org/10.1101/2023.10.22.563504; this version posted November 6, 2023. The copyright holder for this preprint (which was not certified by peer review) is the author/funder, who has granted bioRxiv a license to display the preprint in perpetuity. It is made available under aCC-BY 4.0 International license.

Supplemental Information 2: Right hemisphere data

Compared the left auditory cortex, the right hemisphere SIS activities were less pronounced. Multiple individuals did not show a clear SIS response in the right hemisphere even during the baseline phase in both the adaptation and control experiments (see Fig. S2). In addition, in the adaptation group, we did not observe a significant SIS reduction in adaptation phases in the right auditory cortex, $F(2, 26) = 0.150$, $p = 0.862$, in line with a previous study that found prediction-related SIS effect only in the left hemisphere (Niziolek et al., 2013). We also did not find any significant SIS reduction in the control group's right hemisphere activities, $F(2, 16) = 0.854$, $p = 0.444$. It should be noted that one participant's data in the adaptation group was excluded from analyses because source localization for the right hemisphere response was unreliable.

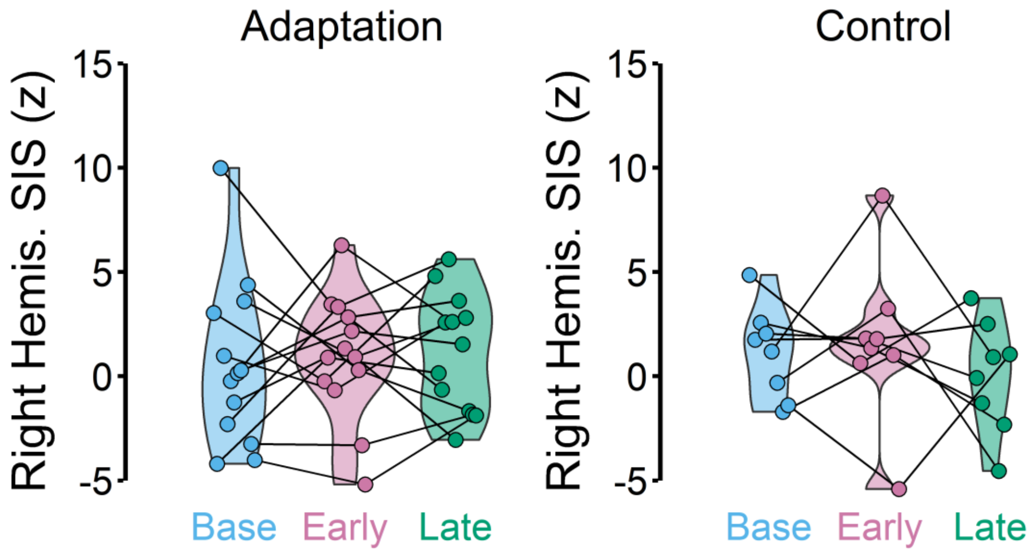

Fig. S2. We did not find a significant SIS effect during the baseline in the right hemisphere. In addition, we did not observe any significant SIS reduction during adaptation.

bioRxiv preprint doi: https://doi.org/10.1101/2023.10.22.563504; this version posted November 6, 2023. The copyright holder for this preprint (which was not certified by peer review) is the author/funder, who has granted bioRxiv a license to display the preprint in perpetuity. It is made available under aCC-BY 4.0 International license.

Supplemental Information 3: Outlier data

One participant's late learning phase SIS response was an outlier, which was imputed in our correlation analysis (see Results). One reason for keeping the data in our analysis was that the behavioral data also clearly indicated that the late learning response was not due to extreme points. As shown in Fig. S3, the participant clearly showed late learning.

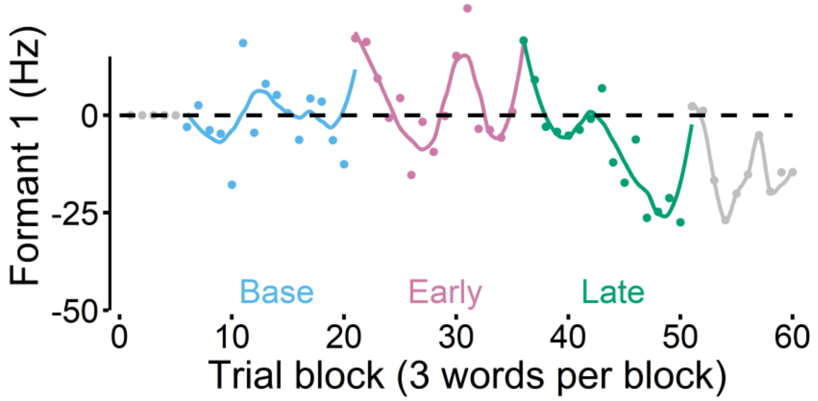

Fig. S3. A participant who had an outlier SIS response (i.e., a large SIS reduction) during the late learning phase also showed large adaptation in the same phase.
